# Dominant Cross-Reactive B Cell Response during Secondary Acute Dengue Virus Infection in Humans

**DOI:** 10.1371/journal.pntd.0001568

**Published:** 2012-03-20

**Authors:** Simona Zompi, Magelda Montoya, Marie O. Pohl, Angel Balmaseda, Eva Harris

**Affiliations:** 1 Division of Infectious Diseases and Vaccinology, School of Public Health, University of California, Berkeley, California, United States of America; 2 Laboratorio Nacional de Virología, Centro Nacional de Diagnóstico y Referencia, Ministerio de Salud, Managua, Nicaragua; Institute of Tropical Medicine (NEKKEN), Japan

## Abstract

The four serotypes of dengue virus (DENV) cause dengue fever (DF) and dengue hemorrhagic fever/dengue shock syndrome (DHF/DSS). Severe disease has been associated with heterotypic secondary DENV infection, mediated by cross-reactive antibodies (Abs) and/or cross-reactive T cells. The role of cross-reactive immunity in mediating enhanced disease versus cross-protection against secondary heterotypic DENV infection is not well defined. A better understanding of the cross-reactive immune response in natural infections is critical for development of safe and effective tetravalent vaccines. We studied the B cell phenotype of circulating B cells in the blood of pediatric patients suspected of dengue during the 2010–2011 dengue season in Managua, Nicaragua (n = 216), which was dominated by the DENV-3 serotype. We found a markedly larger percentage of plasmablast/plasma cells (PB/PCs) circulating in DENV-positive patients as compared to patients with Other Febrile Illnesses (OFIs). The percentage of DENV-specific PB/PCs against DENV-3 represented 10% of the circulating antibody-producing cells (ASCs) in secondary DENV-3 infections. Importantly, the cross-reactive DENV-specific B cell response was higher against a heterotypic serotype, with 46% of circulating PB/PCs specific to DENV-2 and 10% specific to DENV-3 during acute infection. We also observed a higher cross-reactive DENV-specific IgG serum avidity directed against DENV-2 as compared to DENV-3 during acute infection. The neutralization capacity of the serum was broadly cross-reactive against the four DENV serotypes both during the acute phase and at 3 months post-onset of symptoms. Overall, the cross-reactive B cell immune response dominates during secondary DENV infections in humans. These results reflect our recent findings in a mouse model of DENV cross-protection. In addition, this study enabled the development of increased technical and research capacity of Nicaraguan scientists and the implementation of several new immunological assays in the field.

## Author Summary

Dengue is the most common mosquito-borne viral infection of humans, with half the world's population at risk for infection. Four different dengue virus serotypes (DENV-1 to -4) can cause the disease, which can be either inapparent or present with flu-like symptoms (Dengue Fever), also known as “breakbone fever”. In a number of cases, the disease can be more severe and sometimes fatal, with signs of bleeding and vascular leakage leading to shock (Dengue Hemorrhagic Fever/Dengue Shock Syndrome). Severe disease has been associated with secondary sequential DENV infections, i.e., infection with a second DENV serotype different from the serotype causing the first infection. No specific treatment or vaccine is available. Understanding how the human immune response develops during a natural infection can be beneficial for future vaccine studies and trials. B cells are a subset of cells that produce antibodies and are thus essential in the response to natural infections and vaccines. We show here that during secondary DENV infections in humans, the B cell immune response to a previous infecting DENV serotype is stronger than the response against the current infecting serotype. In addition, this study allowed the development of research capacity and implementation of new immunological assays in Nicaragua.

## Introduction

Dengue is the most prevalent mosquito-borne viral disease affecting humans worldwide, mainly encountered in tropical and sub-tropical regions in peri-urban and urban areas, with almost half of the world's population at risk for infection. Dengue is caused by four dengue virus serotypes (DENV-1–4), transmitted by *Aedes aegypti* and *Ae. albopictus* mosquitoes. DENV infection can be asymptomatic or can cause a spectrum of disease, which spans from classical dengue (DF) to more severe forms termed dengue hemorrhagic fever (DHF) and dengue shock syndrome (DSS) [1]. DF is an incapacitating severe flu-like illness that usually resolves spontaneously. The main symptoms include high fever, retro-orbital pain and headache, muscle and joint pain, and rash. DHF/DSS is a potentially fatal form of dengue. DHF is characterized by hemorrhagic manifestations, platelet count ≤100,000 cells/mL; and signs of plasma leakage that may include elevated hematocrit, pleural effusion, ascites, edema, hypoproteinemia and/or hypoalbuminemia. If plasma leakage continues without appropriate fluid resuscitation, DSS can ensue. DSS presents with signs of circulatory failure (narrow pulse pressure or hypotension accompanied by clinical signs of shock) in addition to the signs and symptoms found in DHF. An estimated 500,000 patients require hospitalization each year for DHF/DSS, a large proportion of whom are children [2]. Recently, the WHO developed a new classification of dengue disease that replaces the traditional classification and includes Dengue with or without Warning Signs and Severe Dengue [3]. This new classification has proven to be useful in clinical management of DENV-infected individuals; however, it may be less well-suited for pathogenesis studies [4].

The four DENV serotypes co-circulate in regions like South-East Asia where dengue is hyper-endemic. In contrast, in Nicaragua, one DENV serotype tends to dominate for several years, before being replaced by another serotype, with lower-level co-circulation of other DENV serotypes. DENV-3 has been the dominant serotype circulating in the period 2008 to 2011 in Nicaragua [5]. Prior to this, DENV-2 was the predominant serotype between 1999 and 2002 and again between 2005 and 2007 [5], [6], [7], [8], while DENV-1 predominated between 2002 and 2005 [9]. DENV-4 circulates at a low level in Nicaragua [9].

Although a large proportion of DENV infections remain asymptomatic, epidemiological studies have demonstrated an association between more severe disease and secondary (2°) heterotypic DENV infections with a distinct serotype from the primary (1°) DENV infection [10], [11], [12], [13], [14]. This increase in severity during 2° heterotypic DENV infections has been attributed to antibody (Ab)-dependent enhancement (ADE), where Abs to the 1° infecting serotype bind but do not neutralize the second infecting serotype, instead facilitating an increase in viral uptake by Fcγ-receptor bearing cells [15], [16], [17]. In addition to ADE, cross-reactive T cells, formed during the 1° DENV infection, are over-activated, inducing a “cytokine-storm” syndrome implicated in the pathogenesis of shock syndrome and severe disease [18], [19], [20], [21].

No specific treatment is currently available for dengue, and vaccines trials are in Phase 1 and 2. A better understanding of the immune response developed during natural infections may be beneficial for future vaccine design as well as for defining correlates of protection for the current vaccine trials. Indeed, a balanced and long-lasting T cell, B cell and Ab response against the four serotypes is the goal of an effective tetravalent vaccine. While cross-reactive pre-formed Abs have been implicated in ADE, a cross-reactive B cell and Ab response may be beneficial and protective [22], [23], [24], [25]. In addition, we and others have shown in a mouse model of DENV infection that cross-reactive T cells can be protective [25], [26], [27]. Clearly, in humans, cross-reactive immune responses can be protective, as the majority of 2° DENV infections are asymptomatic or result in mild disease [12].

Different B cell compartments can be identified according to their phenotype, and several B cell subsets circulate in the blood during the acute phase of an infection. Naïve B cells, memory B cells and plasma cells (PCs) are phenotyped by staining with surface markers followed by flow cytometry [28]. During a 1° infection, naïve B cells are stimulated and develop into antigen-specific B cells. These B cells either differentiate into memory B cells, which reside in the secondary lymphoid organs, or into PCs, which secrete antigen-specific Abs. Prior to differentiation into PCs, B cells undergo several cycles of proliferation and differentiate into an intermediate state called plasmablasts (PBs) [28]. Short-lived PCs are active during the acute infection, while long-lived PCs (LLPCs) migrate to the bone marrow and are responsible for long-term humoral immunity [29], [30]. Memory B cells, which retain antigen-specific Abs at their surface, undergo affinity maturation, and only the clones bearing the Abs with the highest affinity survive long-term [31]. This process takes several weeks after the acute infection and continues despite the absence of circulating antigen. Memory B cells are the cells implicated in the antigen recall response and are rapidly activated during a 2° infection [28].

In this study, we analyzed the phenotype of circulating B cells by flow cytometry during the acute phase of infection in patients suspected of dengue presenting to the National Pediatric Reference Hospital, the Hospital Infantil Manuel de Jesús Rivera (HIMJR), in Managua, Nicaragua. The striking increase we observed in the percentage of PB/PCs in DENV-positive patients prompted us to analyze the DENV-specific B cell response by ELISPOT *ex vivo* (representing the circulating PCs at the time of infection) in acute 2° infections, against the current infecting serotype (DENV-3) and against a heterotypic serotype (DENV-2). In addition, we studied the DENV-specific avidity of serum IgG during acute infection and the neutralization capacity of the serum during the acute phase and at 3 months post-onset of symptoms. We found a higher number of cross-reactive DENV-specific PCs, which was associated with greater cross-reactive DENV-specific serum avidity during the acute phase of the infection, suggesting an important role for cross-reactive memory B cells in 2° DENV infections.

## Materials and Methods

### Ethics statement

The protocol for this study was reviewed and approved by the Institutional Review Boards (IRB) of the University of California, Berkeley, and of the Nicaraguan Ministry of Health. Parents or legal guardians of all subjects provided written informed consent, and subjects 6 years of age and older provided assent.

### Study population

This study was performed from August 1, 2010, to January 31, 2011, during the peak of the dengue season in the Nicaraguan National Pediatric Reference Hospital, Hospital Infantil Manuel de Jesús Rivera (HIMJR), located in the capital city of Managua. Inclusion criteria included age between 6 months and 15 years of age, fever or history of fever less than 7 days, and one or more of the following signs and symptoms: headache, arthralgia, myalgia, retro-orbital pain, positive tourniquet test, petechiae, or signs of bleeding. Exclusion criteria included: a) a defined focus other than dengue, b) children weighing less than 8 kg, c) children less than 6 months of age, and d) children 6 years of age and older displaying signs of altered consciousness at the time of recruitment. Patient data such as vital signs, clinical data, and radiographic or ultrasound results were collected on a daily basis by trained medical personnel using a standardized clinical report form until discharge. A blood sample was collected daily for a minimum of three days for Complete Blood Count (CBC) with platelets, blood chemistry, and diagnostic tests for dengue. Between days 14 and 21 after onset of symptoms, a blood sample was collected for convalescent follow-up. In addition, blood samples were collected at 3, 6, 12, and 18 months post-illness onset. At each time-point, plasma and peripheral blood mononuclear cells (PBMCs) were prepared and stored in aliquots at −80°C and liquid nitrogen, respectively.

### Preparation of PBMCs

Daily blood specimens were obtained from patients (average 2.7 samples, range 1–3), along with a convalescent/discharge sample (for 96% of the enrolled patients). Analyzed samples were obtained between 1 and 8 days post-onset of symptoms (mean of 5.6±0.08 days). Five mL of blood were collected in EDTA tubes (Becton-Dickenson, Franklin Lakes, NJ) for children with a body weight greater than 10 kg, and 4 mL were collected for children with a body weight equal or less than 10 kg. The transport temperature (∼28°C), time of sample collection, transport, reception, and processing (total = ∼2.5 hours (h)) were strictly controlled using personal data assistants (PDAs) with barcode scanners. Upon receipt in the National Virology Laboratory, an aliquot of 300 µL was removed for flow cytometry staining (see below), and the remaining 4–5 mL of fresh blood was gently pipetted into a Leucosep tube (Greiner Bio-One) containing 3 mL of Ficoll Histopaque (Sigma), and centrifuged at 500× g for 20 minutes (min) at room temperature. The plasma was removed and frozen in aliquots. The PBMC fraction was collected and transferred to a 15 mL conical tube containing 9 mL of PBS with 2% Fetal Bovine Serum (FBS; Denville Scientific Inc.) and 1% penicillin/streptomycin (Sigma). Cells were washed 3 times in this solution by centrifugation at 500× g for 10 min and resuspended in 10 mL of complete media. Before the third wash, an aliquot of 500 µL was used to obtain a cell count using a hematology analyzer (Sismex XS-1000i). After the third wash, cells were resuspended at a concentration of 10^7^ cells/mL in freezing media consisting of 90% FBS and 10% dimethyl sulfoxide and aliquotted. Average yield was 9.6×10^6^ total cells (3×10^6^ to 17.6×10^6^). Cryovials containing the cell suspension were placed in isopropanol containers (Mr. Frosty, Nalgene) at −80°C overnight and then transferred to liquid nitrogen.

### Laboratory tests

Laboratory confirmation of DENV infection consisted of reverse transcription–polymerase chain reaction (RT-PCR) amplification of viral RNA [32]; isolation of DENV in C6/36 *Aedes albopictus* cells [7]; seroconversion of DENV-specific IgM antibodies as measured by IgM capture enzyme-linked immunosorbent assay (ELISA) [33] between acute-phase and convalescent-phase serum samples; and/or a four-fold or greater increase in total antibody titer, as measured by Inhibition ELISA [9], [34], between paired acute- and convalescent-phase serum samples. Identification of DENV serotype (1–4) was achieved by RT-PCR directed to the capsid gene [32] and/or nonstructural protein 3 gene [35] performed with RNA extracted from serum and/or supernatant of C6/36 cells obtained during virus isolation [36]. Primary DENV infections were defined by an antibody titer by Inhibition ELISA of <10 in acute-phase samples and/or <2,560 in convalescent-phase samples, and secondary DENV infections were defined by an antibody titer by Inhibition ELISA≥10 in acute-phase samples and/or ≥2,560 in convalescent-phase samples [6]. All serologic and virologic assays were performed in the National Virology Laboratory at the National Diagnosis and Reference Center (CNDR) of the Nicaraguan Ministry of Health. All clinical laboratory tests were performed in the Department of Clinical Chemistry at the CNDR or at the clinical laboratory at the Health Center Sócrates Flores Vivas [36] in Managua.

### Viruses and cell lines

DENV was propagated in *Aedes albopictus* C6/36 cells (gift from P. Young, University of Queensland, Australia) in M199 medium (Invitrogen) with 10% FBS at 28°C. Cell supernatants were collected on days 5, 6, 7 and 8 post-infection and either frozen at −80°C directly or after concentration. Concentrated virus was prepared by centrifugation through Amicon filters (50 kDa, 3,250× g for 20 min at 4°C). To prepare antigen for avidity and ELISPOT assays, DENV was cultivated in Vero cells in DMEM medium (Invitrogen) with 10% FBS at 37°C and 5% CO_2_. Cell supernatants were collected on days 5, 6, 7 and 8 post-infection, clarified and concentrated by ultracentrifugation (26,000× g for 2 h at 4°C) and resuspended in TNE (Tris buffer, NaCl and EDTA) or PBS. DENV-2 (strain N172, passage 2) and DENV-3 (strain N7236, passage 3) are clinical strains from two Nicaraguan patients isolated in the National Virology Laboratory in Managua, Nicaragua, and passaged minimally in our laboratory. Virus titers were obtained by plaque assay on baby hamster kidney cells (BHK21, clone 15) as previously described [37]. Raji-DC-SIGN-R cells (gift from B. Doranz, Integral Molecular, Philadelphia, PA) were grown in RPMI-1640 medium (Invitrogen) with 5% FBS at 37°C in 5% CO_2_ for use in neutralization assays [38], [39].

### Flow cytometry

On days 1, 2, and 3 of hospitalization, 300 ul of fresh whole blood was collected. Red blood cells were lysed using 1× RBC lysis buffer (eBioscience). Cells were then blocked in 5% Normal Rat serum (Jackson ImmunoResearch Inc.) before staining. Cells were stained with anti-CD138 (MI-15) or anti-HLA-DR FITC (G46-6), anti-CD20 PECy7 (2H7), anti-CD27 PE (O323), and anti-CD38 PECy-5 (HIT2). For the analysis of marginal zone (MZ) B cells, cells were stained with anti-IgD FITC (IA6-2), anti-CD20 PECy7, anti-CD27 PE, and anti-IgM PECy-5 (G20-127). Finally, cells were fixed in 2% paraformaldehyde. Samples were analyzed on a 4-color flow cytometer (Epics XL, Beckman-Coulter). Results were analyzed using FlowJo software, version 7.2.5 (TreeStar Software). All flow cytometric analysis was performed in the National Virology Laboratory at the CNDR in Managua.

### ELISPOT assay

To quantify the number of DENV-specific PCs, frozen PBMCs from day 6 post-onset of symptoms were thawed and analyzed by ELISPOT *ex vivo* [40]. Ninety-six-well filter plates were first coated with 10 µg/well 4G2 monoclonal antibody (MAb) (mouse, pan-DENV) overnight at 4°C and then blocked for 2 h at 37°C with RPMI-1640 medium plus 10% FBS. Viruses DENV-2 N172 or DENV-3 N7236 prepared from infected Vero cells by ultracentrifugation were UV-inactivated for 10 min and then incubated with the plates at a dilution of 1∶25 in PBS to capture the virus. To detect the total number of IgG-secreting cells (including both DENV-specific and non-specific ASCs), wells were coated with donkey anti-human IgG (10 µg/mL, Jackson ImmunoResearch Inc.). Virus-coated and anti-IgG-coated plates were incubated for 5–6 h with PBMCs to allow formation of Ab-antigen complexes (anti-DENV Abs with DENV and total IgG with anti-IgG). Duplicate samples of 1×10^5^ PBMCs per well (for wells containing DENV antigen) and 3×10^4^ per well (for wells containing anti-human IgG) were plated in the first well, and four 2-fold dilutions were distributed in the subsequent wells. After the incubation period, cells were removed, and plates were washed and incubated with biotinylated anti-human IgG Ab overnight (1/1,000, Jackson ImmunoResearch Inc.), followed by Streptavidin-Alkaline Phosphatase (AP, Vector Inc.) and BCIP/NBT substrate (Vector Inc.). Resulting spots, representing DENV-specific Ab-producing B cells or total IgG Ab-producing cells, were counted by visual inspection using an inverted microscope. Control wells were coated with 4G2 MAb and PBS with no virus. For each sample, spots counted in the control wells were subtracted from the spots counted in the test wells coated with DENV-specific antigen. ELISPOT responses were considered to be positive if the number of spots was >200 spots/10^6^ PBMCs for total IgG.

### Neutralization assay

Serum samples from the acute phase (day 6 post-onset of symptoms) and 3 months post-onset of symptoms were heat-inactivated at 56°C for 20 min and then diluted using eight 3-fold dilutions, beginning at 1∶10 and extending to 1∶21,870. Neutralization was assessed by flow cytometry using a reporter (GFP) system with pseudo-infectious DENV reporter virus particles (RVPs) [39]. DENV RVP production (DENV-1, Western Pacific 74; DENV-2, S16803; DENV-3, CH53489; DENV-4, TVP360; gift from B. Doranz, Integral Molecular) was performed in 293TREx cell lines as described [38], [39]. Supernatants containing RVPs were harvested, passed through 0.45-µm filters, aliquotted, and stored at −80°C. For all experiments, DENV RVPs were rapidly thawed from cryopreservation in a 37°C water bath and placed on ice for use in neutralization assays. DENV RVPs in RPMI-1640 complete medium were pre-incubated with an equal volume of serially diluted serum samples for 1 h at room temperature with slow agitation. Raji DC-SIGN-R cells were added to each well at a density of 40,000 cells per well, followed by incubation at 37°C in 5% CO_2_ for 48 h. Cells were subsequently fixed in 2% paraformaldehyde and analyzed for the percentage of cells expressing GFP by flow cytometry (Becton-Dickinson LSRII). The percent infection for each serum dilution was calculated, and the raw data was expressed as percent infection versus log_10_ of the reciprocal serum dilution. The data were fitted to a sigmoidal dose-response curve, using Prism (GraphPad Prism 5.0 Software) to determine the titer of antibody that achieved a 50% reduction in infection (50% neutralization titer, NT_50_). The NT_50_ titer is expressed as the reciprocal of the serum dilution. Maximum infection was determined in the absence of serum.

### Avidity assay

Serum avidity was measured using a modified ELISA protocol with urea washes [25], [41], [42]. Supernatant from Vero cells infected with DENV-2 N172 and DENV-3 N7236 was ultracentrifuged (26,000× g for 2 h at 4°C) to prepare concentrated virus. Viruses were UV-inactivated for 10 min, plated in carbonate buffer overnight in a flat-bottom 96-well plate, washed, and then blocked with PBS-T (PBS with 0.1% Tween-20) containing 5% nonfat dry milk. Wells were incubated for 1 h with serum samples from 1° or 2° DENV infections diluted in blocking buffer. Convalescent samples (day 14 to 21 post-onset of symptoms) were used for the analysis of 1° DENV infections, while acute samples (day 6 post-onset of symptoms) were used for the analysis of 1° DENV infections. The plates were washed for 10 min with different concentrations of urea (6 M urea for primary DENV cases and 9 M urea for secondary DENV cases) before adding the secondary biotin-conjugated Ab (donkey anti-human IgG) and streptavidin-AP conjugate. Finally, PnPP substrate was added to the wells, and optical density (OD) values were measured at 405 nm using KC Junior software. Background levels were measured in wells that were treated with normal human serum. For each plate, background was subtracted, and percentage of IgG bound was calculated by dividing the adjusted OD after urea washes by the adjusted OD after PBS.

### Statistical analysis

Non-parametric analyses using the two-sided Wilcoxon Rank Sum test were used for pairwise comparisons, and the Mann-Whitney test was used for non-paired analysis. The Spearman test was used to examine correlations. Calculations were performed in GraphPad Prism 5.0 software.

## Results

### Study participants

Between August 1, 2010, and January 31, 2011, 216 patients were enrolled for suspected dengue at the National Pediatric Reference Hospital, HIJMR. Twelve patients were excluded from analysis; one patient dropped out of the study after enrollment and 11 patients had an undetermined dengue diagnostic result. Overall, 204 patients were followed up and their characteristics are shown in Table 1. One hundred and thirty patients (63.7%) were laboratory-confirmed as dengue-positive. Among these, 75 (36.8%) were 1° and 55 (63.2%) were secondary 2° DENV infections (Table 1). Serotype identification was achieved in 86.2% of dengue-positive cases, with 108 of 112 (96.4%) confirmed as DENV-3 infections. Of note, the severity of disease was relatively low in this season, with 32 (26.4%) dengue-positive cases classified as DHF/DSS [1]. Prior to circulation of DENV-3 as the dominant serotype in 2008–2010 [5], DENV-2 was the predominant circulating serotype in Nicaragua between 1999 and 2002 and again between 2005 and 2007 [6], [7], [8], while DENV-1 predominated between 2002 and 2005 [9]. Thus, children with secondary DENV infections were most probably previously infected with DENV-1, DENV-2, or both.

**Table 1:** Characteristics of patients enrolled in the hospital-based study during the 2010–2011 dengue season, Managua, Nicaragua.

	Total participants (n = 204*)
**Sex**	
Male	101 (49.5%)
Female	103 (50.5%)
**Age (years)**	
<1	7 (3.4%)
1–4	44 (21.6%)
5–9	82 (40.2%)
10–14	69 (33.8%)
>14	2 (1.0%)
Median (range)	7.9 yrs (7 months-15.8 yrs)
**Final result**	
Dengue-positive	130 (63.7%)
Other Febrile Illness	74 (36.3%)
**Immune Status**	
Primary DENV infection	75 (36.8%)
Secondary DENV infection	55 (63.2%)
**Serotype**	
DENV-1	3 (2.3%)
DENV-2	1 (0.8%)
DENV-3	108 (83.1%)
Unknown	18 (13.8%)
**Severity**	
DF	98 (75.4%)
DHF	30 (23.1%)
DSS	2 (1.5%)

*: Out of 216 participants enrolled, 1 participant dropped out and 11 participants were excluded for indeterminate dengue diagnostic results.

### Increased percentage and numbers of PB/PCs in DENV-positive cases

**Figure 1:**
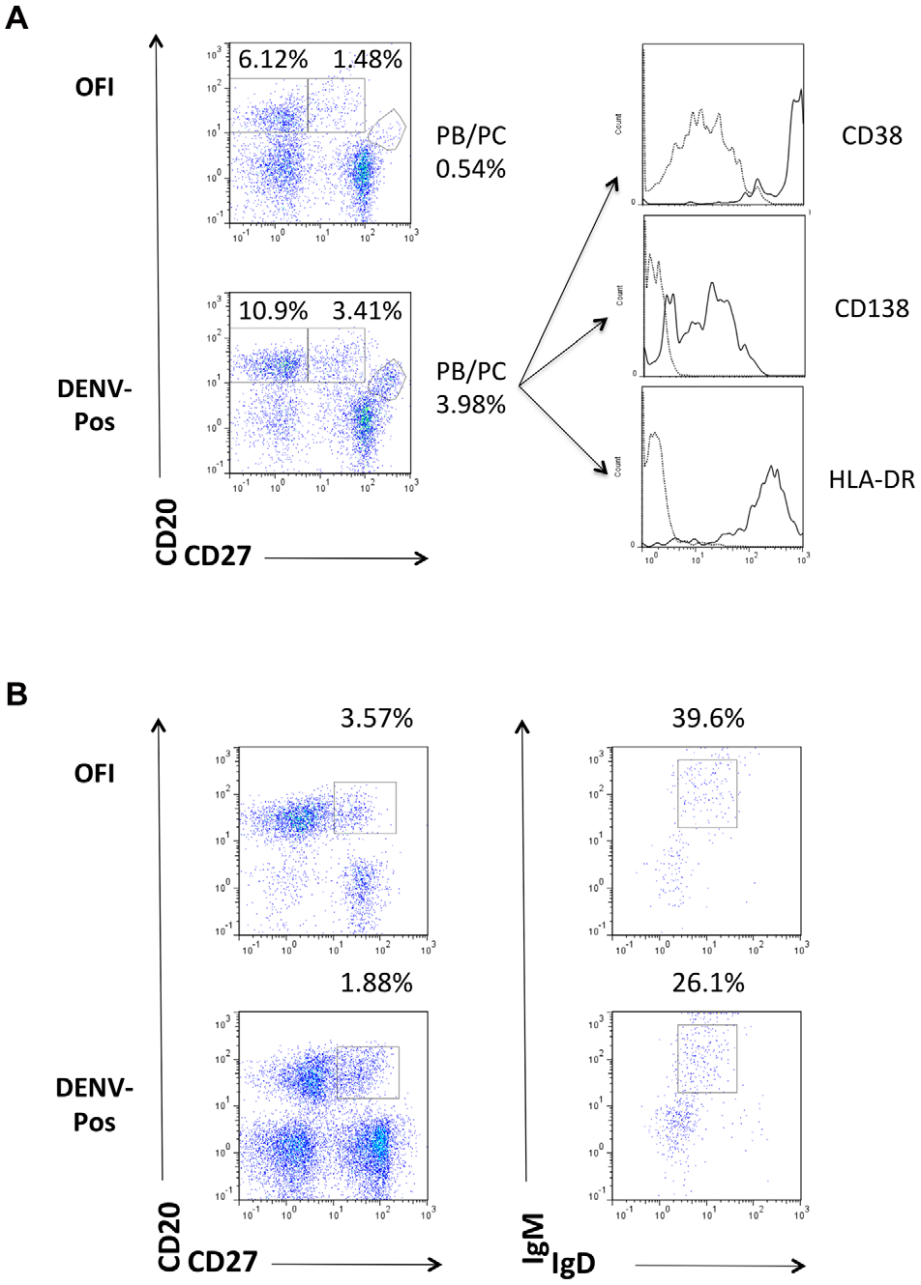
Flow cytometry staining of B cell subsets in peripheral blood of DENV-infected patients. **A.** Phenotype of circulating B cells in patients suspected of DENV infection. Whole blood was collected during the first 3 days of hospitalization and stained directly with anti-CD138 or anti-HLA-DR FITC, anti-CD20 PECy7, anti-CD27 PE, and anti-CD38 PECy-5. Cells were gated on the lymphocyte population, and B cell sub-populations were discriminated using anti-CD20 and anti-CD27 MAbs. Naïve B cells are CD20^+^CD27^−^, memory B cells are CD20^+^CD27^+^, and PB/PCs are CD20^low^CD27^high^. Histograms represent levels of CD38, CD138 and HLA-DR expression on PB/PCs; dark grey line, positive stained sample; light grey line, negative unstained control sample. One representative flow cytometry staining (from day 5 post-onset of symptoms) is shown out of 22 OFI and 38 DENV-positive cases processed by flow cytometry. **B.** Phenotype of MZ B cells in patients suspected of DENV infection. For MZ B cell analysis, cells were processed as in Figure 1A and stained with anti-IgD FITC, anti-CD20 PECy7, anti-CD27 PE, and anti-IgM PECy-5. Cells were gated on the lymphocyte population, and B cell sub-populations were identified using anti-CD20 and anti-CD27 MAbs. MZ B cells are CD20^+^CD27^+^IgD^+^IgM^+^. One representative flow cytometry staining (from day 2 post-onset of symptoms) is shown out of 23 OFIs and 31 DENV-positive cases processed by flow cytometry.

Fresh whole blood collected during the first three days of hospitalization in the HIMJR was stained with MAbs and analyzed by flow cytometry in order to phenotype the B cells circulating at the time of infection. Dengue diagnostic (RT-PCR) results were obtained within 24 h after hospital admission. B cells from all cases were phenotyped on day 1, while B cells from all dengue-positive cases and one out of every five OFI cases were phenotyped on all three days. This staining allowed us to distinguish between naïve B cells (CD20^+^CD27^−^), memory B cells (CD20^+^CD27^+^) and PB/PCs (CD20^low^CD27^high^) (Figure 1A). In addition, among the memory B cells, the marginal zone (MZ) B cell subset was analyzed (IgD^+^IgM^+^) (Figure 1B). As expected, the PB/PCs expressed high levels of CD38, which is a marker of cell activation, and variable levels of CD138, which is a cell surface marker found only on PCs. In addition, this population expressed high levels of HLA-DR, indicating activation of these cells (Figure 1A).

**Figure 2:**
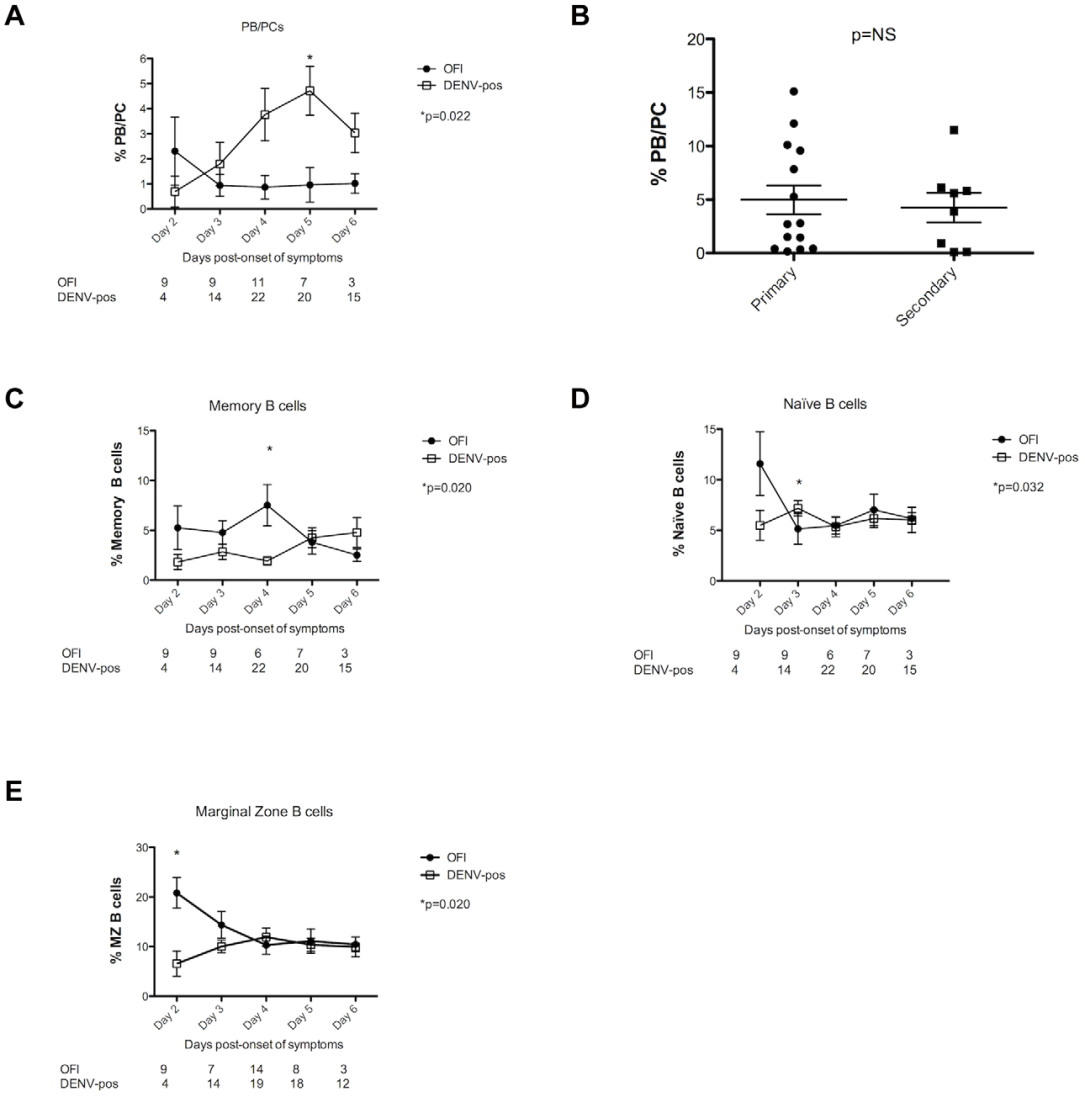
Increased percentage of PB/PCs in peripheral blood of DENV-infected patients. **A.** Percentage of PB/PCs circulating in the blood of patients suspected of DENV infection. Mean and SE of the percentage of PB/PCs among total lymphocytes plotted according to day post-onset of symptoms. As the same patient may have had up to 3 samples processed, the patient may be represented more than once over time. The number of samples processed is shown below the graph. The percentage of PB/PCs increased over time in DENV-positive samples and peaked at day 5 post-onset of symptoms. Statistical analysis was performed using the Mann-Whitney test, and a significant difference in percentage of PB/PC between OFIs and DENV-positive cases was found at day 5 post-onset of symptoms. The p-value is shown below the symbol legend. **B.** Percentage of PB/PCs at day 5 post-onset of symptoms in 1° and 2° DENV infections. Statistical analysis was performed using the Mann-Whitney test, and no significant difference in percentage of PB/PCs was found at day 5 post-onset of symptoms between 1° and 2° DENV infections. n = 14 for 1° DENV infections; n = 8 for 2° DENV infections. **C.** Percentage of memory B cells circulating in the blood of patients suspected of DENV infection. Mean and SE of the percentage of memory B cells plotted according to day post-onset of symptoms. As the same patient may have had up to 3 samples processed, the patient may be represented more than once over time. The number of samples processed is shown below the graph. The evolution over time of the percentage of memory B cells in OFIs and DENV-positive cases is similar. Statistical analysis was performed using the Mann-Whitney test, and a significant difference between OFIs and DENV-positive cases was found on day 4 post-onset of symptoms. The p-value is shown below the symbol legend. **D.** Percentage of naïve B cells circulating in the blood of patients suspected of DENV infection. Mean and SE of the percentage of naïve B cells plotted according to day post-onset of symptoms. As the same patient may have had up to 3 samples processed, the patient may be represented more than once over time. The number of samples processed is shown below the graph. The evolution over time of the percentage of naïve B cells in OFIs and DENV-positive cases is similar. Statistical analysis was performed using the Mann-Whitney test, and a significant difference between OFIs and DENV-positive cases was found on day 3 post-onset of symptoms. The p-value is shown below the symbol legend. **E.** Percentage of MZ B cells circulating in the blood of patients suspected of DENV infection. Mean and SE of the percentage of MZ B cells plotted according to day post-onset of symptoms. As the same patient may have had up to 3 samples processed, the patient may be represented more than once over time. The number of samples processed is shown below the graph. The evolution over time of the percentage of MZ B cells in OFIs and DENV-positive cases is similar. Statistical analysis was performed using the Mann-Whitney test, and a significant difference between OFIs and DENV-positive cases was found on day 2 post-onset of symptoms. The p-value is shown below the symbol legend.

The percentages of different B cell subsets were then analyzed over time. While no increase in percentage of PB/PCs over time was observed in OFI cases, this percentage increased and peaked on day 5 post-onset of symptoms in DENV-positive. On day 5, a significant increase in percentage of PB/PCs was found in DENV-positive patients as compared to OFI cases (mean DENV-positive = 4.72±0.97% *vs.* mean OFI = 0.96±0.69%, p = 0.022) (Figure 2A). Of note, among DENV-positive patients, no statistical difference in percentage of PB/PCs was found at day 5 post-onset of symptoms between 1° and 2° infections (mean 1° = 4.99±1.35% *vs.* mean 2° = 4.25±1.38%, p = 0.76) (Figure 2B) or between DF and DHF/DSS cases (mean DF = 4.42±1.23% *vs.* mean DHF/DSS = 5.50±1.51%, p = 0.48) (data not shown). A lower percentage of memory B cells was found on day 4 post-onset of symptoms in DENV-positive cases (mean DENV-positive = 1.93±0.42% *vs.* mean OFI = 7.52±2.07%, p = 0.020), but no clear increase over time was seen in either of the two populations (Figure 2C). A slightly higher percentage of naïve B cells was noted on day 3 post-onset of symptoms in DENV-positive cases (mean DENV-positive = 7.16±0.76% *vs.* mean OFI = 5.14±1.52%, p = 0.032), but again no clear increase over time was seen in either population (Figure 2D). A significantly higher percentage of MZ B cells was found on day 2 post-onset of symptoms in OFI cases (mean DENV-positive = 6.57±2.55% *vs.* mean OFI = 20.82±3.09%, p = 0.020), but no significant differences were found at later time-points (Figure 2E). These data correlate with data on absolute numbers of B cells calculated based on the number of total lymphocytes (Figure S1). Of note, despite a higher number of total lymphocytes in OFI, the numbers of PB/PCs are greater in DENV-positive patients when compared to OFI between days 4 and 6 post-onset of symptoms.

### Increased numbers of cross-reactive DENV-specific PCs on day 6 post-onset of symptoms in secondary DENV infections

**Figure 3:**
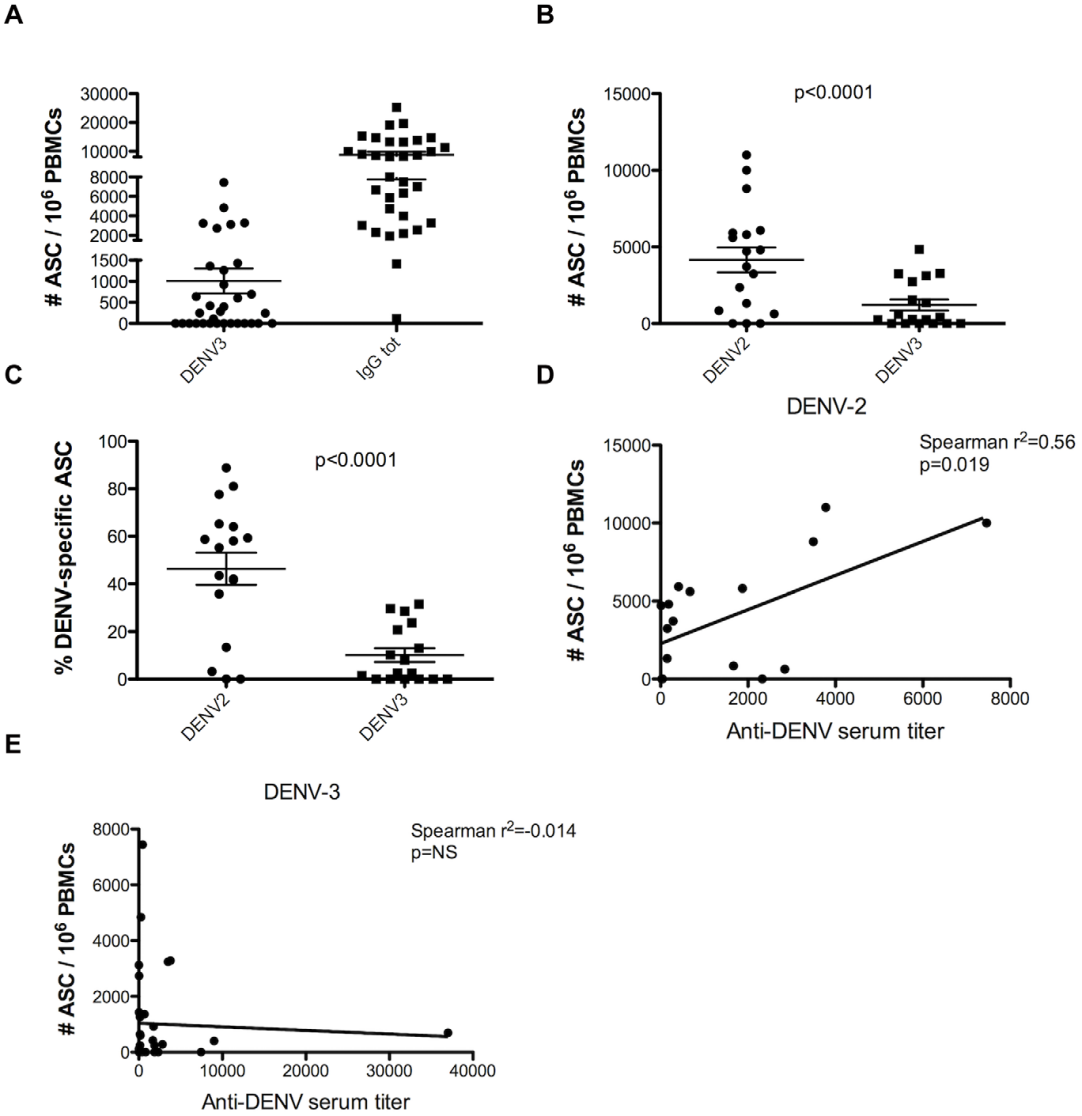
Increased cross-reactive DENV-specific PCs during acute secondary DENV infections. **A.** Number of DENV-3-specific ASCs and total ASCs circulating in the peripheral blood during 2° DENV-3 infections. PBMCs from day 6 post-onset of symptoms from 33 patients enrolled with 2° DENV-3 infections were prepared and frozen in liquid nitrogen. Thawed PBMCs were plated on 96-well filter plates coated with either DENV-3 virus, to detect DENV-3-specific ASCs, or anti-human IgG (positive control), to detect the total number of ASCs. **B.** Number of DENV-2-specific ASCs and DENV-3-specific ASCs circulating in peripheral blood during 2° DENV-3 infections. PBMCs from day 6 post-onset of symptoms from 17 patients enrolled with 2° DENV-3 infections were prepared and frozen in liquid nitrogen. Thawed PBMCs were plated on 96-well filter plates coated with either DENV-2 virus, to detect DENV-2-specific ASCs, or DENV-3 virus, to detect DENV-3-specific ASCs. Statistical analysis was performed by Wilcoxon Rank Sum test, and a significant difference in the number of DENV-2-specific ASCs vs. DENV-3-specific ASCs was found. The p-value is shown in the graph. **C.** Percentage of DENV-2-specific ASCs and DENV-3-specific ASCs circulating in peripheral blood during 2° DENV-3 infections, in relation to total ASCs. The percentage of DENV-2-specific and DENV-3-specific ASCs was calculated by dividing the number of DENV-2- and DENV-3-specific ASCs by the number of total ASCs. PBMCs were processed as in Figure 3C. Statistical analysis was performed by Wilcoxon Rank Sum test, and a significant difference in the percentage of DENV-2-specific ASCs vs. DENV-3-specific ASCs was found. The p-value is shown in the graph. **D.** Correlation between DENV-2-specific PCs and total anti-DENV antibody titer during the acute phase of DENV-3 2° infections. The number of DENV-2-specific PCs/10^6^ PBMCs measured by *ex vivo* ELISPOT at day 6 post-onset of symptoms is plotted against the total anti-DENV antibody titer measured during the acute phase of the infection (n = 17). Statistical analysis was performed using the Spearman test. A weak positive statistical significant correlation was found between the two variables. The r^2^ and the p-values are shown in the graph. **E.** Correlation between DENV-3-specific PCs and total anti-DENV antibody titer. The number of DENV-3-specific PCs/10^6^ PBMCs measured by *ex vivo* ELISPOT at day 6 post-onset of symptoms is plotted against the total anti-DENV antibody titer measured during the acute phase of the infection (n = 33). Statistical analysis was performed using the Spearman test. No statistical significant correlation was found between the two variables. The r^2^ and the p-values are shown in the graph.

The characteristics of the patients with 2° DENV infections enrolled during the study are shown in Table 2. Among the 55 cases, only confirmed DENV-3-positive cases were processed by ELISPOT to measure the number of DENV-specific PCs circulating in the peripheral blood during the acute phase (day 6 post-onset of symptoms). Concentrated preparations of virions from clinical isolates of DENV-2 and DENV-3 from Nicaragua, minimally passaged in the laboratory, were used as antigen in order to match as closely as possible the virus to which the patients were exposed. Of 33 cases with detectable ASCs, DENV-3-specific PCs represented 11.5% of the total ASC/10^6^ PBMCs (mean DENV-3-specific ASC = 1,008±295 ASC/10^6^ PBMCs and mean total ASC = 8,783±1,028 ASC/10^6^ PBMCs) (Figure 3A).

**Table 2:** Characteristics of patients with secondary DENV infection with PBMCs processed by ELISPOT.

	Secondary infections (n = 55)	DENV-3 ELISPOT (n = 33)*	DENV-2 and DENV-3 ELISPOT (n = 17)**
**Sex**			
Male	27	17	8
Female	28	16	9
**Age (years)**			
<1	0	0	0
1–4	0	0	0
5–9	20	15	7
10–14	31	16	9
>14	4	2	0
Median (range)	10.5 yrs (5.5–15.8 yrs)	10.2 yrs (5.5–14.9 yrs)	9.8 yrs (5.7–13.2 yrs)
**Serotype**			
DENV-1	1	0	0
DENV-2	1	0	0
DENV-3	47	33	17
Unknown	6	0	0
**Severity**			
DF	36	21	12
DHF	17	11	4
DSS	2	1	1

*: Subset of secondary DENV infections among the 55 patients for which only DENV-3-specific ASC were assayed by ELISPOT.

**: Subset of secondary DENV infections among the 33 patients for which both DENV-2- and DENV-3-specific ASC were assayed by ELISPOT.

The median age of patients experiencing secondary DENV infection was 10.5 years, with a range of 5.5 to 15.8 years. According to epidemiological data regarding the DENV serotypes that have been circulating recently in Nicaragua [6], [7], [8], [9], [43], these children could have been previously infected by DENV-1 and/or DENV-2. As these are pediatric cases, the volume of blood drawn is restricted and thus the availability of PBMCs was limited. Therefore, only a subset of samples was processed using a second DENV serotype, in this case DENV-2, in addition to DENV-3 as antigen (Table 2). DENV-2 was chosen to represent a cross-reactive, heterotypic serotype to which patients in the study were likely to have been exposed. A significantly higher number of DENV-2-specific ASC was found in these 2° DENV infections when compared to the number of DENV-3-specific ASC (mean DENV-2 ASC = 4,402±823 ASC/10^6^ PBMCs vs. mean DENV-3 ASC = 1,129±373 ASC/10^6^ PBMCs; p<0.0001) (Figure 3B). DENV-2-specific ASC represented on average 46±7% of the total ASC circulating at the time of infection, compared to 10±3% DENV-3-specific ASC (p<0.0001) (Figure 3C). Overall, these data show an increase in DENV-specific PCs during acute 2° DENV infections, with a greater increase in cross-reactive PCs that are specific to a previous infecting serotype rather than the current infecting serotype. A positive correlation was found between the titer of total DENV-specific Abs as measured by Inhibition ELISA and the number of DENV-2-specific PCs during acute infection, while no correlation was found with the number of DENV-3-specific PCs (Figure 3D and E). This result suggests that the anti-DENV specific Abs are mostly produced by the cross-reactive PCs during an acute 2° DENV infection.

### Increased cross-reactive DENV-specific serum avidity in secondary DENV infections

**Figure 4:**
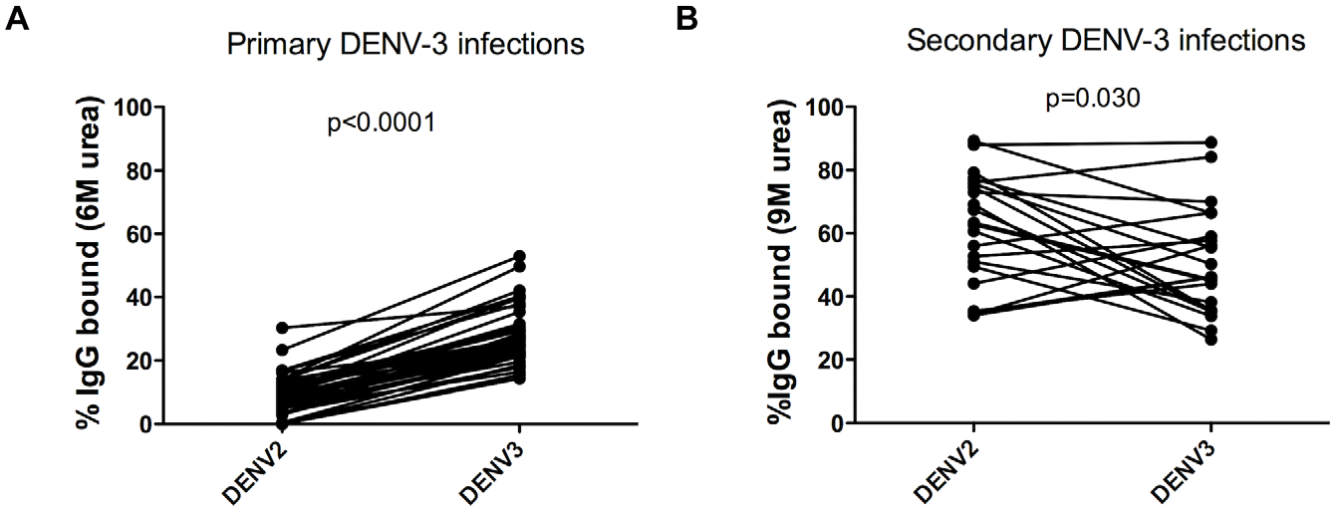
Increased cross-reactive DENV-specific IgG serum avidity during acute secondary DENV infections. **A.** DENV-specific IgG serum avidity at convalescence in 1° DENV-3 infections. Convalescent serum (day 14 to 21 post-onset of symptoms) from a subset (n = 42) of 1° DENV-3 infections was tested for DENV-specific avidity using a modified ELISA with 6 M urea washes. The same serum samples were tested in parallel against DENV-2 and DENV-3 Nicaraguan viruses. Statistical analysis was performed by Wilcoxon Rank Sum test, and a significant difference in DENV-2-specific IgG serum avidity vs. DENV-3-specific IgG serum avidity was found. The p-value is shown above the graph. **B.** DENV-specific IgG serum avidity at day 6 post-onset of symptoms in 2° DENV-3 infections. Acute serum from day 6 post-onset of symptoms from a subset (n = 18) of 2° DENV-3 infections was tested for DENV-specific avidity using a modified ELISA with 9 M urea washes. The same serum samples were tested in parallel against DENV-2 and DENV-3 Nicaraguan viruses. Statistical analysis was performed by Wilcoxon Rank Sum test, and a significant difference in DENV-2-specific IgG serum avidity vs. DENV-3-specific IgG serum avidity was found. The p-value is shown above the graph.

In order to measure IgG serum avidity, we used a modified ELISA with urea washes [25], [41], [44]. The same clinical viral isolates from Nicaragua that were used in the ELISPOT assays were used in the avidity assay. To validate the assay using samples and virus from Nicaragua, we tested a subset of 42 1° DENV-3 cases from the 2010 hospital study. As the amount of IgG is low during the acute phase of 1° infections, we used serum samples from the convalescent phase (day 14 to 21 post-onset of symptoms). The serum avidity of these samples was measured against both DENV-2 and DENV-3. As expected, higher avidity was found against the infecting DENV serotype, DENV-3, with a low level of cross-reactivity against DENV-2 (mean % IgG bound to DENV-3 = 27.7±1.4% vs. mean % IgG bound to DENV-2 = 9.4±0.9%; p<0.0001) (Figure 4A).

We then measured the DENV-specific serum avidity during the acute phase of 2° DENV-3 infections (day 6 post-onset of symptoms). The same subset of samples that was processed for DENV-2 and DENV-3 ELISPOT was processed by the avidity assay. As shown in Figure 4B, the cross-reactive serum avidity against DENV-2 was significantly higher than the homotypic serum avidity against DENV-3 (mean % IgG bound to DENV-2 = 61.3±3.7% vs. mean % IgG bound to DENV-3 = 50.7±3.6%; p = 0.030). Overall, these data show a greater cross-reactive DENV-specific IgG serum avidity as compared to homotypic DENV-specific IgG serum avidity during the acute phase of 2° DENV infections.

### Cross-reactive DENV-specific neutralization profile of serum in secondary DENV infections

**Figure 5:**
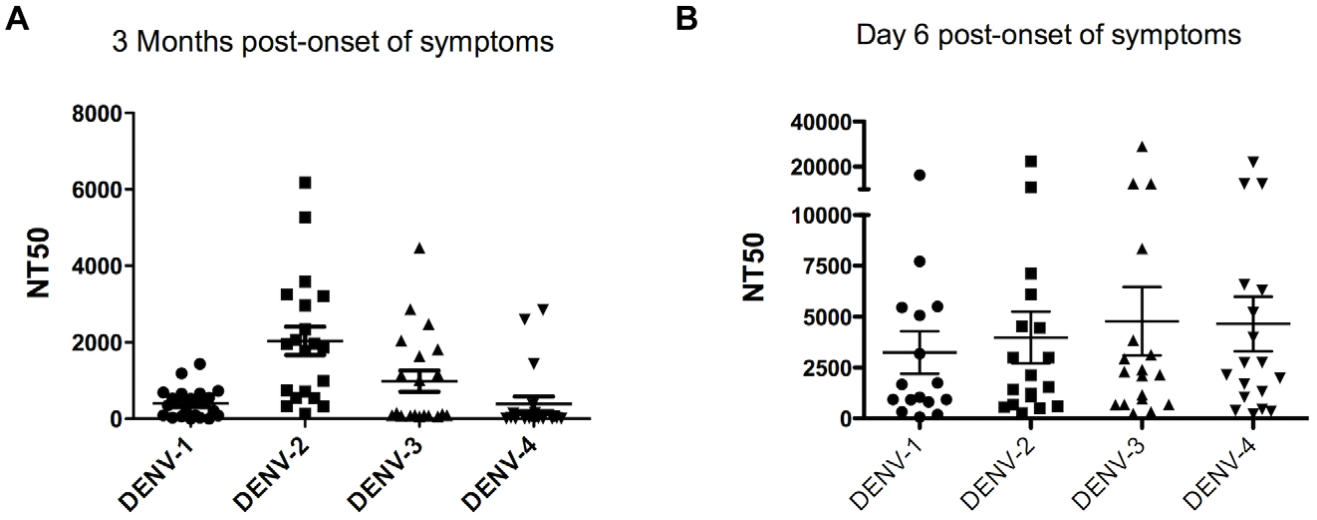
Cross-reactive DENV-specific serum neutralization during secondary DENV infections. **A.** DENV-specific neutralization of the serum at 3 months post-onset of symptoms in 2° DENV-3 infections. Longitudinal serum samples, obtained 3 months post-onset of symptoms from a subset (n = 20) of 2° DENV-3 infections, were analyzed in a flow cytometry-based neutralization assay. Briefly, serial dilutions of the serum samples were incubated with RVPs containing the GFP gene and C-prM/M-E from the 4 different DENV serotypes for one h, and then incubated with Raji-DC-SIGN-R cells for 48 h. The percentage of infection, as defined by expression of GFP in the infected cells, was detected by flow cytometry. The NT_50_, calculated using Prism software (see Material and Methods), represents the serum dilution at which 50% neutralization of infection is achieved. Statistical analysis was performed by Wilcoxon Rank Sum test to compare the NT_50_ of each heterotypic serotype (DENV-1, DENV-2 and DENV-4) to the infecting serotype (DENV-3). No significant difference was found. **B.** DENV-specific neutralization of serum at day 6 post-onset of symptoms in 2° DENV-3 infections. Samples obtained at day 6 post-onset of symptoms from a subset (n = 20) of 2° DENV-3 infections were analyzed in a flow cytometry-based neutralization assay to measure the NT_50_ as described in the legend to Figure 5A. Statistical analysis was performed by Wilcoxon Rank Sum test to compare the NT_50_ of each heterotypic serotype (DENV-1, DENV-2 and DENV-4) to the infecting serotype (DENV-3). No significant difference was found.

Finally, we measured the DENV-specific neutralization capacity of patient serum against the 4 DENV serotypes using an RVP flow cytometry-based neutralization assay. The same subset of samples that was processed for DENV-2 and DENV-3 ELISPOTs was processed by the neutralization assay. The NT_50_ titer of 2° DENV-3 infections at 3 months post-onset of symptoms is shown in Figure 5A. The NT_50_ titer was high not only against DENV-3 (mean 986±276), the current infecting serotype, but also against DENV-2 (mean 2039±371). The NT_50_ against DENV-1 (mean 404±91) and DENV-4 (mean 390±192) were lower but detectable. Thus, after 2° DENV infections, a broad cross-reactive neutralization response develops against the 4 serotypes, consistent with previous reports.

In addition, we measured the NT_50_ titer of these same samples during the acute phase of the infection at day 6 post-onset of symptoms. As expected, NT_50_ titers were higher during the acute phase when compared to the 3-month samples. The NT_50_ titer was high not only against DENV-3 (mean 4783±1687), the current infecting serotype, but also against DENV-2 (mean 3979±1274), DENV-1 (mean 3244±1049), and DENV-4 (mean 4654±1342). Thus, as at 3 months post-onset of symptoms, we found a broadly cross-reactive response to all 4 serotypes during the acute phase of the infection (Figure 5B). Of note, no statistical significant difference was found between anti-DENV-2 and anti-DENV-3 NT_50_ titers, either during the acute phase or at the 3-month time-point.

## Discussion

In this study, we used flow cytometry to phenotype the B cell components circulating at the time of DENV infection, using fresh whole blood in Nicaragua. In addition, we measured the number of DENV-specific PCs during acute infection by ELISPOT using Nicaraguan virus preparations as antigen. Finally, we measured both the DENV-specific IgG serum avidity and neutralization capacity of the serum against different serotypes of DENV. Overall, we show that a large number of PB/PCs circulate during DENV infection when compared to OFIs, both during 1° and 2° DENV infections. We find a strikingly higher number of DENV-specific PCs and serum IgG avidity directed to a heterotypic DENV serotype (DENV-2) as opposed to the current infecting serotype (DENV-3). Overall, we show that a cross-reactive B cell response dominates during the acute phase of 2° human DENV infections.

A large percentage of PB/PCs circulate in the blood of DENV-infected children during the acute phase of infection, in both 1° and 2° DENV infections, as compared to children with OFIs. Of note, the amount of PB/PCs does not vary with age [45]. The percentage of PB/PCs circulating in the blood peaked at day 5 post-onset of symptoms. While we would have expected a high percentage of PB/PCs in both DENV-infected and OFI patients, the difference was marked and might point to either a stronger B cell response during DENV infections when compared to OFIs or to a difference between the time-points after infection at which the samples were collected in DENV-positive cases versus OFI cases. The definitive diagnosis of OFI cases is not known; however, possible differential diagnoses include influenza, rickettsiosis, and leptosporosis, among others. In an effort to define the possible viral etiology of OFIs, we analyzed DENV-negative cases using viral microarrays followed by deep sequencing and detected *Human Herpesvirus 6* sequence and sequences related to other *Herpesviridae and Circaviridae* [46]. The course of disease of the OFIs, which may be different from dengue illness, and the fact that PB/PCs circulate in the blood for only a short period of time as compared to other B cell components [47] may explain the differences in percentage of PB/PCs between these two groups. In addition, certain viruses, like influenza and measles, are known to depress the immune system [48]; thus, some OFI patients may experience decreased proliferation of B cells either directly or secondarily due to decreased proliferation of T-helper cells, resulting in reduced numbers of PB/PCs. Of note, no difference in percentage of PB/PCs circulating in blood was noted when comparing 1° and 2° DENV infections.

In contrast to PB/PCs, which circulate in the blood during a narrow time-window, the number of memory B cells circulating in the blood increases later during infection [47]. We observed an increase over time of memory B cells in DENV-infected patients, whereas this subset of cells decreased in OFI patients. Marginal zone (MZ) B cells are IgM^+^ “memory” B cells that have been implicated in the response against encapsulated bacteria, such as *S. pneumoniae* [49]. These cells are implicated in T-cell-independent immune responses and despite the presence of IgM at their surface, they present hypermutated immunoglobulin receptors [50], [51], [52]. Recently, highly neutralizing IgM^+^ MAbs have been generated from individuals infected by influenza [53], and these MAbs have been shown to arise from the MZ B cell population [53]. We did not find a clear difference in the percentage of this population between the two groups. Thus, this subset of cells may not play a role during DENV infections.

In order to further characterize the PB/PCs circulating during acute DENV infection, we measured the number of DENV-specific PCs at day 6 post-onset of symptoms by *ex vivo* ELISPOT, i.e., without any stimulation of the PBMCs. First, we found that DENV-3-specific PCs constitute a substantial proportion (∼10%) of total ASCs in the blood of patients with a 2° DENV-3 infection. Among the patients experiencing a 2° DENV-3 infection, a subset of samples were processed by ELISPOT against both DENV-2 and DENV-3 viruses. Interestingly, we found a higher number of PCs specific for the non-infecting serotype (DENV-2) when compared to the currently infecting serotype (DENV-3). These DENV-2-specific PCs made up 46% of the total ASCs. These findings were associated with the IgG serum avidity data, where higher serum avidity was detected against DENV-2 as compared to DENV-3. Thus, during an acute 2° DENV infection, cross-reactive PCs and cross-reactive Abs responsible for the higher avidity increase more than homotypic PCs and homotypic Abs directed to the current infecting serotype. In addition, a positive correlation between the total anti-DENV Ab titer was found only with DENV-2 specific PCs but not with DENV-3 specific PCs, consistent with other reports [44]. Thus, the increased number of anti-DENV Abs circulating during a 2° infection may be induced by cross-reactive PCs, and this rise in Ab titer is associated with an increased IgG serum avidity against a heterotypic serotype. These findings support the initial concept of “original antigenic sin” in dengue immunopathogenesis, whereby the humoral immune response in a secondary DENV infection is stronger to the prior infecting serotype [54], [55].

These data are in accordance with our findings in our mouse model of sequential DENV infection, where we observed an increase in PCs, memory B cells, and highly avid Abs against the previous infecting serotype rather than against the current infecting serotype [25]. These data are also in accordance with recently published human data, which show an increase in cross-reactive memory B cells and cross-reactive serum avidity during the acute phase of 2° DENV infection in a population of DENV-infected children in Thailand [44]. These two sets of data are complementary, as we measured the number of DENV-specific PCs *ex vivo* (plated directly for ELISPOT without prior *in vitro* stimulation) during acute infection, while Mathew et al. [44] measured the number of memory B cells obtained from PBMCs polyclonally stimulated *in vitro*. Overall, these two studies suggest that the increase in cross-reactive PCs during an acute 2° DENV infection is mediated by the cross-reactive memory B cells formed during a previous infection with a different serotype.

Neutralization assays during the acute phase and at 3 months post-onset of symptoms show a broadly cross-reactive response against the four serotypes of DENV, as previously described [56]. Thus, there appears to be no association during 2° DENV infection between neutralization capacity of the serum and the number of circulating DENV-specific PCs or increased DENV-specific serum avidity. Direct correlation between neutralization capacity of serum and serum avidity has not been shown thus far during DENV infection. In fact, it was found that no direct correlation exists between neutralization capacity and affinity of anti-DENV MAbs [57](K. Williams and E. Harris, unpublished data). In addition, in our mouse model of sequential DENV infection, we demonstrated an uncoupling of the neutralization and avidity responses during 2° DENV infections, with a higher DENV-specific avidity against the 1° infecting serotype and an increased neutralization capacity of the serum against the 2° infecting serotype [25]. Further analysis of 1° and 2° serum samples, including samples from patients enrolled in our Nicaraguan or other cohort studies for which the 1° infecting serotype is known, are needed to further investigate this question in humans.

While other groups have used recombinant proteins for avidity and ELISPOT assays [44], we used viral particles as antigen, prepared from Nicaraguan clinical viral isolates. Previous data have shown that human anti-DENV and anti-West Nile Virus (WNV) Abs bind to the viral prM/M protein and to sites on the envelope (E) protein or on several E monomers on the virion that are not preserved in the recombinant E formulation [58], [59], [60]. Thus, we preferred to use whole viral particles in our assays to better approximate the viral antigen seen by the immune response *in vivo*. In addition, the use of clinical viral isolates from Nicaragua represents the most relevant viral strains.

The 2010–2011 dengue season in Nicaragua was characterized by low disease severity, with only 30 (23.1%) cases of DHF and 2 cases (1.5%) of DSS in our study. We did not find any difference in the number of DENV-specific PCs or in serum avidity during the acute infection between DF and DHF/DSS, but differences may exist in more severe cases. Further analyses of the B cell response during subsequent seasons with greater severity are warranted to study such associations. In addition, disease severity can be influenced by the serotype-specific sequence of infections and the time interval between sequential DENV infections [61], [62], [63], issues that are better addressed using samples from prospective cohort studies. A separate study of a prior DENV-2 epidemic in Managua revealed a trend towards decreased serum avidity in more severe DSS cases when compared to DF and DHF cases (M.O. Pohl, S. Zompi and E. Harris, unpublished data) using both a urea-based ELISA and a virus competition ELISA [55]. More refined analysis of the serum avidity by surface plasmon resonance may be more sensitive, and such studies are currently underway.

This study has several strengths. Given our established mouse model of DENV infection and disease, we can study the immune response in parallel in mice and humans. The mouse model allows a more complete mechanistic approach, e.g., allowing the investigation of the role of the different immune components during DENV infections [25], while the human studies extend the relevance of the findings to the clinical situation. For the first time, B cell- and Ab-based assays, including ELISPOTs and urea-based ELISAs, were carried out using viral particles purified from clinical isolates from the field as antigen. Using this type of antigen, prepared by propagating the virus in mammalian Vero cells, enables as close an approximation to the *in vivo* situation as possible. Finally, the flow cytometry was performed at the NVL/CNDR in Managua, Nicaragua. Although this limited the analysis to several four-color panels due to the cytometer available at the CNDR, it allowed analysis of fresh whole blood from children enrolled in the hospital-based dengue study. Importantly, establishing this assay in Nicaragua increased the research and technical skills of NVL personnel, which is complemented by our program of continuous training of Nicaraguan scientists at UC Berkeley in relevant scientific and technical areas. In-country use of the cytometer also resulted in continuous maintenance of the machine, which is now being used for additional projects, such as flow cytometry-based neutralization assays for serological investigation of DENV infection over time.

One of the main limitations of this study was the low level of severity observed during the 2010–2011 season, which did not allow correlations between the number of DENV-specific PCs circulating during acute infection and disease severity to be performed. The use of samples from future more severe epidemics will be useful in investigating this question. In addition, the previous infecting serotype(s) of the 2° DENV infections hospitalized in this study is unknown. The use of samples from cohort studies, in which patients are followed prospectively over time, will allow an improved analysis of the serotype-cross-reactive response initially observed in this study.

Overall, we have shown that during DENV infection, a high number of PB/PCs circulate in the blood and that during 2° DENV infection, the DENV-specific PCs are mostly cross-reactive and likely arise from memory B cells formed during previous heterotypic infections. This is associated with an increase in cross-reactive DENV-specific IgG serum avidity. The assays used in this study were either performed at the NVL in Managua, Nicaragua, or at UC Berkeley in collaboration with a researcher from Nicaragua who was trained in ELISPOT and avidity ELISA assays, thus increasing research capacity of Nicaraguan scientists. In addition, these assays were performed using clinical viral isolates from Nicaragua, better approximating the *in vivo* situation in humans. Lastly, these assays should be useful in the characterization of the humoral immune response induced by candidate dengue vaccines.

## Supporting Information

Figure S1 **Absolute number of B cell sub-populations circulating in the blood of DENV-suspected cases at day 6 post-onset of symptoms.** **A.** Absolute number of lymphocytes in the blood of patients suspected of DENV infection. Mean and SE of the number of lymphocytes were plotted according to day post-onset of symptoms. As the same patient may have had up to 3 samples processed, the patient may be represented more than once over time. The number of samples processed is shown below the graph. The number of lymphocytes increased over time in DENV-positive and OFI samples. Statistical analysis was performed using the Mann-Whitney test, and a significant difference in number of lymphocytes between OFI and DENV-positive cases was found between day 2 and day 5 post-onset of symptoms. The p-value is shown below the symbol legend. **B.** Absolute number of PB/PCs in the blood of patients suspected of DENV infection. Mean and SE of the number of lymphocytes were plotted according to the day post-onset of symptoms. As the same patient may have had up to 3 samples processed, the patient may be represented more than once over time. The number of samples processed is shown below the graph. Statistical analysis was performed using the Mann-Whitney test, and no significant difference in number of PB/PCs was found between DENV-positive and OFI cases, with a trend towards higher numbers in DENV-positive cases between days 3 and 6 post-onset of symptoms. **C.** Absolute number of memory B cells circulating in the blood of patients suspected of DENV infection. Mean and SE of the number of memory B cells were plotted according to day post-onset of symptoms. As the same patient may have had up to 3 samples processed, the patient may be represented more than once over time. The number of samples processed is shown below the graph. The number of memory B cells decreases over time in OFI cases, while it increases over time in DENV-positive cases. Statistical analysis was performed using the Mann-Whitney test, and a significant difference between OFI and DENV-positive cases was found between days 2 and 4 post-onset of symptoms. The p-value is shown below the symbol legend. **D.** Absolute number of naïve B cells circulating in the blood of patients suspected of DENV infection. Mean and SE of the number of naïve B cells were plotted according to the day post-onset of symptoms. As the same patient may have had up to 3 samples processed, the patient may be represented more than once over time. The number of samples processed is shown below the graph. The evolution over time of the number of naïve B cells in OFI and DENV-positive cases is similar. Statistical analysis was performed using the Mann-Whitney test, and a significant difference between OFI and DENV-positive cases was found on day 2 post-onset of symptoms. The p-value is shown below the symbol legend.
(DOC)
