# Supplementary figures and images for: Dominant Cross-Reactive B Cell Response during Secondary Acute Dengue Virus Infection in Humans
Source: PLoS Negl Trop Dis. 2012 Mar 20;6(3):e1568. doi: 10.1371/journal.pntd.0001568 (PMC3308930; doi:10.1371/journal.pntd.0001568)

**
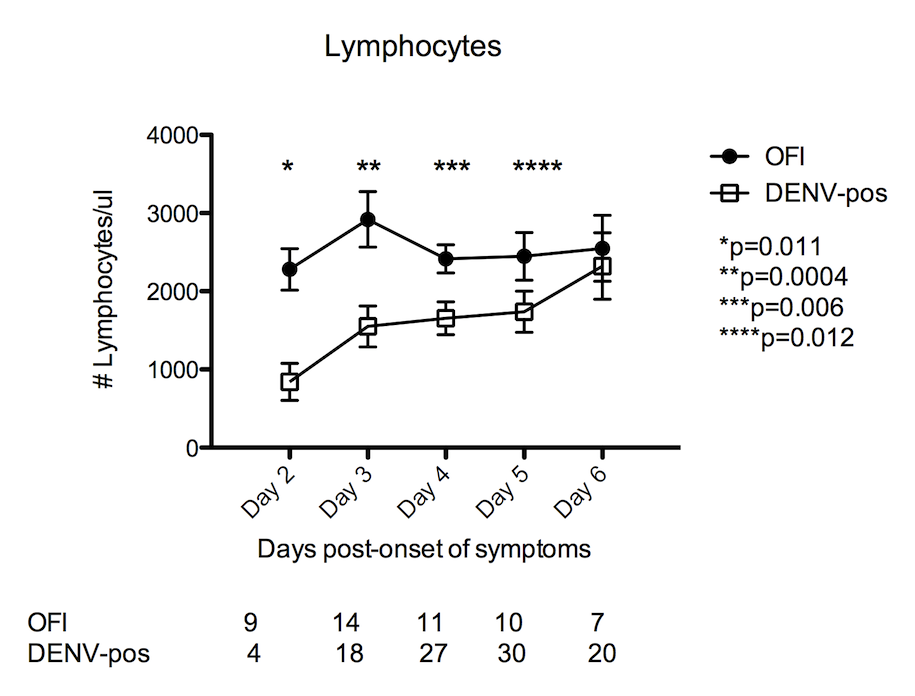

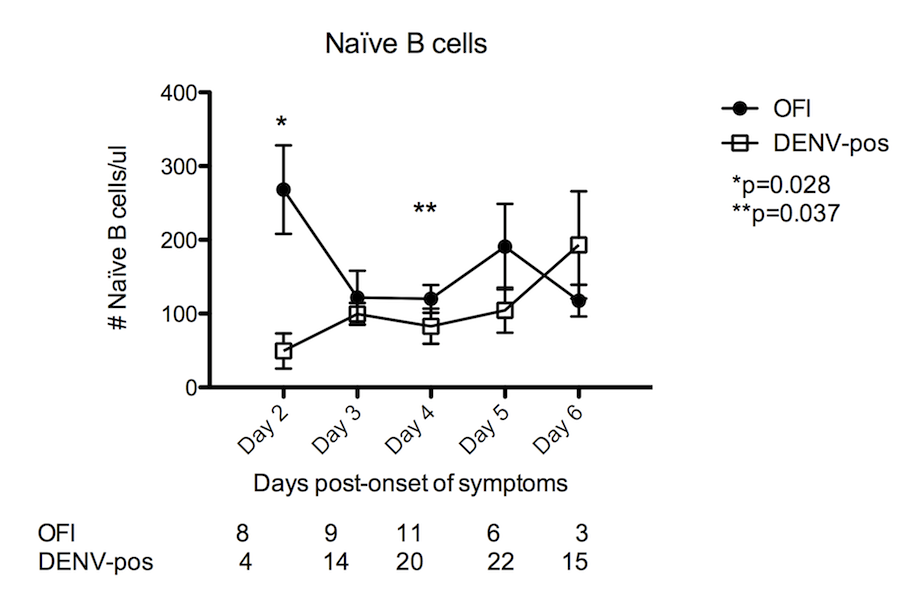

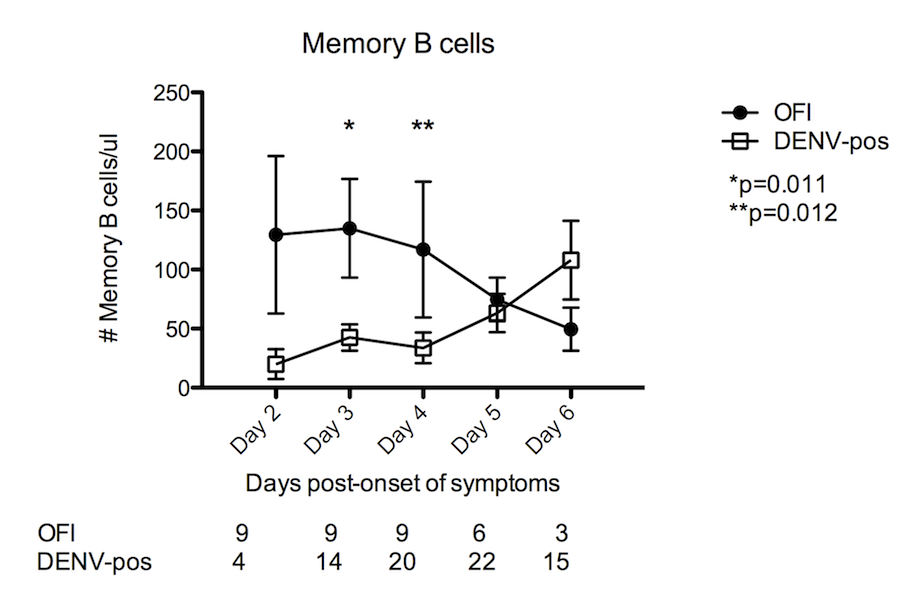

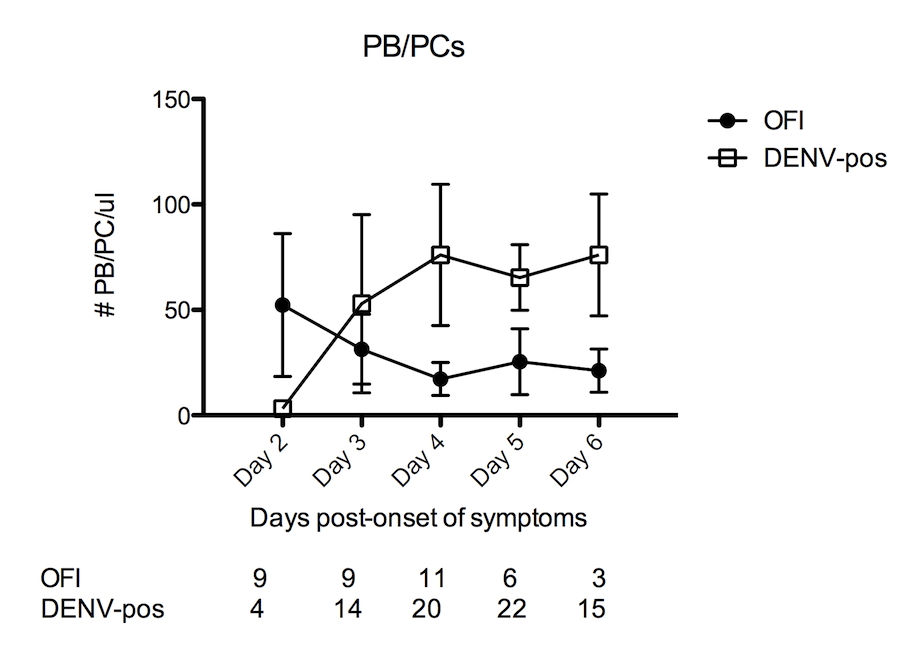
**

**A.**

**D.**

**C.**

**B.**

Supplement: Figure S1 — Absolute number of B cell sub-populations circulating in the blood of DENV-suspected cases at day 6 post-onset of symptoms. A. Absolute number of lymphocytes in the blood of patients suspected of DENV infection. Mean and SE of the number of lymphocytes were plotted according to day post-onset of symptoms. As the same patient may have had up to 3 samples processed, the patient may be represented more than once over time. The number of samples processed is shown below the graph. The number of lymphocytes increased over time in DENV-positive and OFI samples. Statistical analysis was performed using the Mann-Whitney test, and a significant difference in number of lymphocytes between OFI and DENV-positive cases was found between day 2 and day 5 post-onset of symptoms. The p-value is shown below the symbol legend. B. Absolute number of PB/PCs in the blood of patients suspected of DENV infection. Mean and SE of the number of lymphocytes were plotted according to the day post-onset of symptoms. As the same patient may have had up to 3 samples processed, the patient may be represented more than once over time. The number of samples processed is shown below the graph. Statistical analysis was performed using the Mann-Whitney test, and no significant difference in number of PB/PCs was found between DENV-positive and OFI cases, with a trend towards higher numbers in DENV-positive cases between days 3 and 6 post-onset of symptoms. C. Absolute number of memory B cells circulating in the blood of patients suspected of DENV infection. Mean and SE of the number of memory B cells were plotted according to day post-onset of symptoms. As the same patient may have had up to 3 samples processed, the patient may be represented more than once over time. The number of samples processed is shown below the graph. The number of memory B cells decreases over time in OFI cases, while it increases over time in DENV-positive cases. Statistical analysis was performed using the Mann-Whitney test, an [file pntd.0001568.s001.doc]
